# 4′-(4-Fluoro­phen­yl)-1′-methyl­dispiro­[indane-2,2′-pyrrolidine-3′,2′′-indane]-1,3,1′′-trione methanol hemisolvate

**DOI:** 10.1107/S1600536813009987

**Published:** 2013-04-17

**Authors:** Mohamed Ashraf Ali, Elumalai Manogaran, Tan Soo Choon, Mohd Mustaqim Rosli, Ibrahim Abdul Razak

**Affiliations:** a Institute for Research in Molecular Medicine, Universiti Sains Malaysia, 11800 USM, Penang, Malaysia; b Faculty of Pharmaceutical Science, UCSI (University College Sedaya International) University, Kuala Lumpur, Malaysia; c X-ray Crystallography Unit, School of Physics, Universiti Sains Malaysia, 11800 USM, Penang, Malaysia

## Abstract

The asymmetric unit of the title compound, C_29_H_24_FNO_5_·0.5CH_3_OH, contains two independent mol­ecules and a one methanol solvent mol­ecule. The methanol mol­ecule is O—H⋯O hydrogen bonded to one of the independent mol­ecules. The pyrrolidine rings in both mol­ecules adopt half-chair conformations, while the cyclo­pentane rings within the indane groups are in flattened envelope conformations, with the spiro C atoms forming the flaps. The benzene rings of the indane ring systems form a dihedral angle of 35.06 (7)° in one independent mol­ecule and 31.16 (8)° in the other. The fluoro-substituted benzene ring forms dihedral angles of 65.35 (6) and 85.87 (7)° with the indane group benzene rings in one mol­ecule, and 72.78 (8) and 77.27 (8)° in the other. In each mol­ecule, a weak intra­molecular C—H⋯O hydrogen bond forms an *S*(6) ring motif. In the crystal, weak C—H⋯O, C—H⋯N and C—H⋯F hydrogen bonds link the mol­ecules into a three-dimensional network.

## Related literature

For background to compounds with anti­tubercular activity, see: Ali *et al.* (2011 ▶). For related structures, see: Wei *et al.* (2011 ▶, 2012 ▶). For hydrogen-bond motifs, see: Bernstein *et al.* (1995 ▶). For ring conformations, see: Cremer & Pople (1975 ▶). For the stability of the temperature controller used in the data collection, see: Cosier & Glazer (1986 ▶).
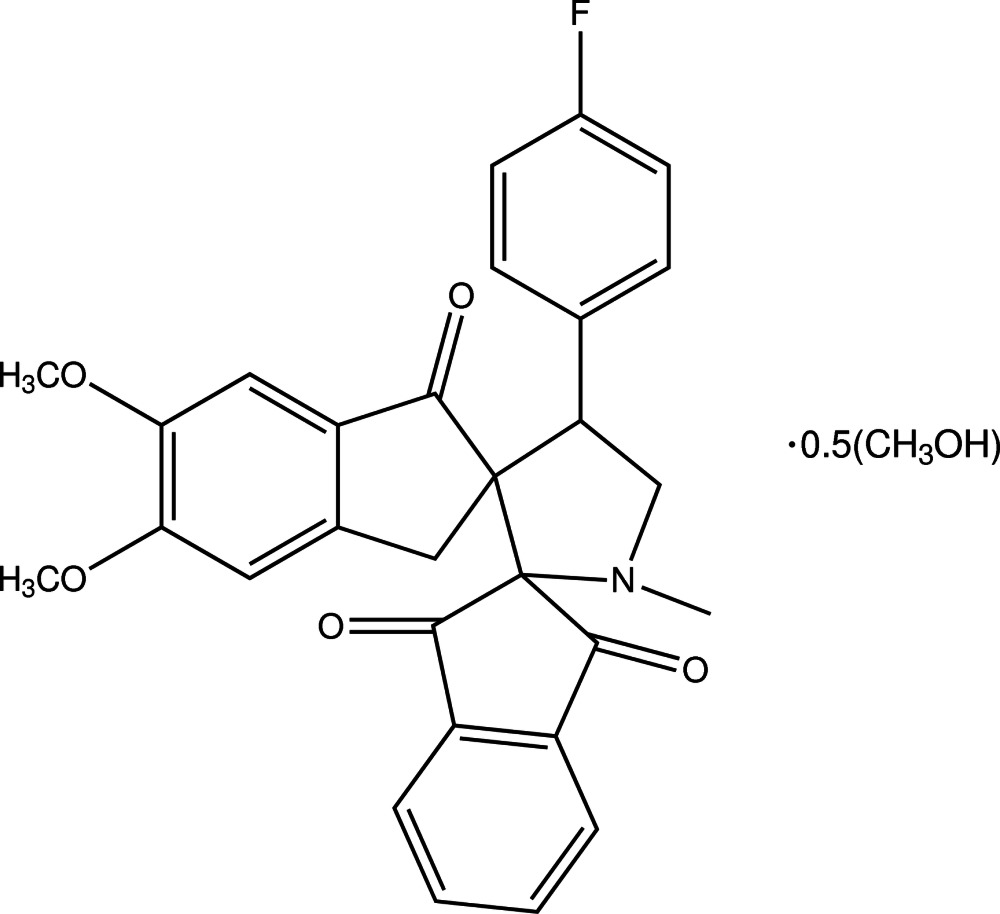


## Experimental

### 

#### Crystal data

C_29_H_24_FNO_5_·0.5CH_4_O
*M* *_r_* = 501.52
Monoclinic, 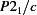

*a* = 14.6385 (6) Å
*b* = 12.5099 (6) Å
*c* = 26.2017 (10) Å
β = 92.645 (1)°
*V* = 4793.1 (4) Å^3^
*Z* = 8
Mo *K*α radiation
μ = 0.10 mm^−1^
*T* = 100 K
0.44 × 0.21 × 0.15 mm

#### Data collection

Bruker APEX DUO CCD area-detector diffractometer
Absorption correction: multi-scan (*SADABS*; Bruker, 2009 ▶) *T*_min_ = 0.957, *T*_max_ = 0.985
53390 measured reflections
14094 independent reflections
10283 reflections with *I* > 2σ(*I*)
*R*_int_ = 0.046

#### Refinement

*R*[*F* ^2^ > 2σ(*F* ^2^)] = 0.049
*wR*(*F* ^2^) = 0.147
*S* = 1.04
14094 reflections
678 parameters
H atoms treated by a mixture of independent and constrained refinement
Δρ_max_ = 0.40 e Å^−3^
Δρ_min_ = −0.27 e Å^−3^

### 

Data collection: *APEX2* (Bruker, 2009 ▶); cell refinement: *SAINT* (Bruker, 2009 ▶); data reduction: *SAINT*; program(s) used to solve structure: *SHELXTL* (Sheldrick, 2008 ▶); program(s) used to refine structure: *SHELXTL*; molecular graphics: *SHELXTL*; software used to prepare material for publication: *SHELXTL* and *PLATON* (Spek, 2009 ▶).

## Supplementary Material

Crystal structure: contains datablock(s) I, global. DOI: 10.1107/S1600536813009987/lh5596sup1.cif

Structure factors: contains datablock(s) I. DOI: 10.1107/S1600536813009987/lh5596Isup2.hkl

Additional supplementary materials:  crystallographic information; 3D view; checkCIF report

## Figures and Tables

**Table 1:** Hydrogen-bond geometry (Å, °)

*D*—H⋯*A*	*D*—H	H⋯*A*	*D*⋯*A*	*D*—H⋯*A*
O6—H1*O*6⋯O4*A*	0.97 (3)	1.99 (3)	2.9346 (18)	164 (3)
C8*A*—H8*AA*⋯O5*A*	0.99	2.43	3.088 (2)	124
C8*B*—H8*BA*⋯O5*B*	0.99	2.38	3.0960 (19)	128
C26*A*—H26*A*⋯O5*A* ^i^	0.95	2.54	3.2430 (18)	131
C27*A*—H27*A*⋯N1*B* ^ii^	0.98	2.42	3.337 (2)	155
C28*A*—H28*A*⋯O2*B* ^iii^	0.98	2.50	3.3478 (19)	145
C28*B*—H28*F*⋯O6^iv^	0.98	2.46	3.360 (2)	153
C30—H30*B*⋯F1*A* ^v^	0.98	2.53	3.307 (2)	136

Symmetry codes: (i) 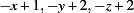; (ii) 
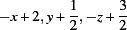
; (iii) 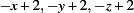; (iv) 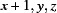; (v) 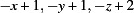.

**Fig. 1.**
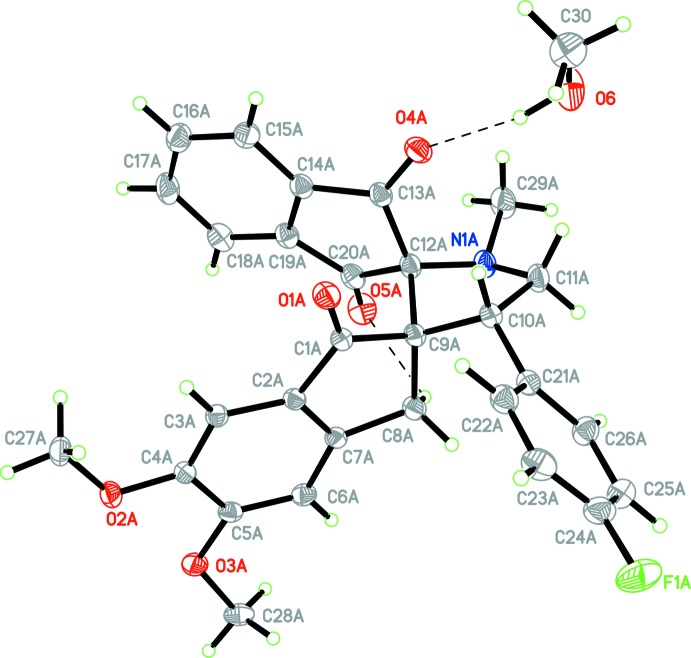
Molecule A of the title compound, showing 50% probability displacement ellipsoids. The dashed lines indicate hydrogen bonds. The solvent molecule is also shown.

**Fig. 2.**
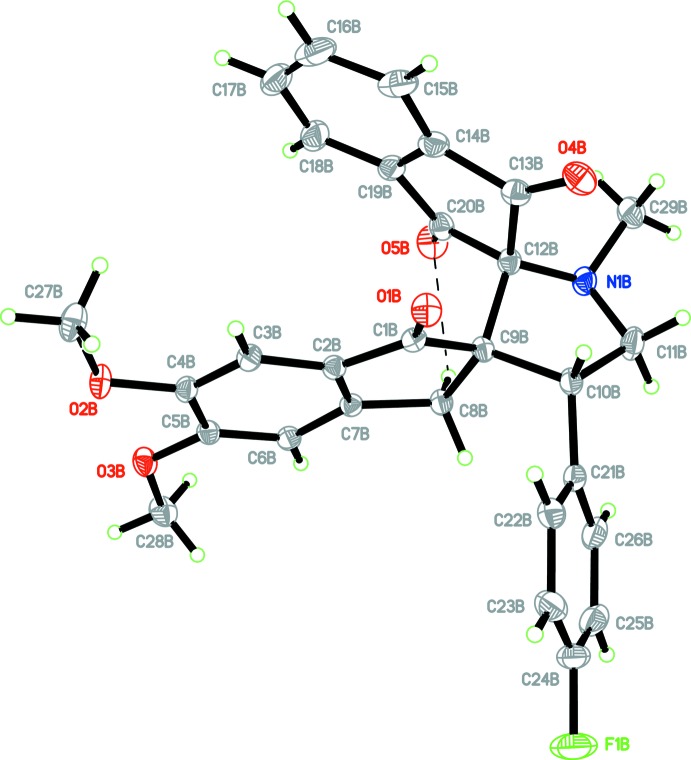
Molecule B of the title compound, showing 50% probability displacement ellipsoids. The dashed line indicates a weak hydrogen bond.

**Fig. 3.**
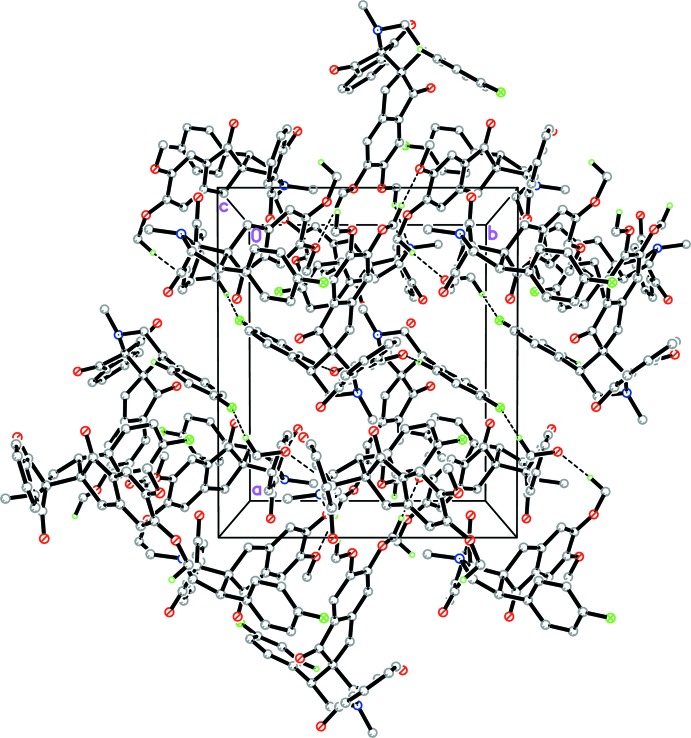
The crystal packing of (I). Dashed lines indicate hydrogen bonds. H atoms not involved in the hydrogen bond interactions have been omitted for clarity.

## supplementary crystallographic information

### Comment

Tuberculosis (TB) remains a global health problem and has infected about one third of the world population. No new drugs have been discovered for the past 40 years and therefore new anti-TB agents are desperately needed (Ali *et al.*, 2011). As part of our ongoing search for novel heterocyclic compounds with antitubercular activity (Wei *et al.*, 2011, 2012) the crystal structure of the title compound (I) has been determined.

The asymmetric unit of the title compound (Fig. 1) contains two crystallographically independent molecules and a molecule of methanol (Fig. 1). In both molecules, the intramolecular interactions of C8A—H8AA···O5A and C8B—H8BA···O5B (Table 1) form an S(6) ring motif (Fig. 2) (Bernstein *et al.* 1995). The pyrrolidine ring for both molecules A and B adopt a half-chair conformation with the puckering parameters Q = 0.4612 (16) Å, φ = 127.36 (19)° for molecule A and Q = 0.4332 (16) Å, φ = 125.2 (2)° for molecule B. In both molecules, A and B, the cyclopentane rings (C1—C2/C7—C9 & C12—C14/C19—C20) within the indane moiety (C1—C9 & C12—C20) form flattened envelope conformations (C9 and C12 at the flap) with the puckering parameters Q = 0.1587 (16) Å, φ 146.2 (6)° and Q = 0.1910 (16) Å, φ = 175.0 (5)° for molecule A and Q = 0.1338 (15)å, φ = 155.5 (6)° and Q = 0.2232 (16) Å, φ = 173.6 (4)° for molecule B.

In each molecule, the benzene rings of the indane ring systems form dihedral angles of 35.06 (7) ° [C2A-C7A/C14A-C19A] and 31.16 (8)° [C2B-C7B/C14B-C19B] with each other. The fluoro-substituted benzene ring forms diedral angles of 65.35 (6)° [C14A-C19A/C21A-C26A] and 85.87 (7)° [C2A-C7A/C21A-C26A] with the indane group benzene rings of one molecule and 72.78 (8)° [C14B-C19B/C21B-C26B] and 77.27 (8)° [C2B-C7B/C21B-C26B] in the other.

In the crystal, molecules are connected by weak C—H···O^i, iii, iv^, C—H···N^ii^ and C—H···F^v^ (Table 1) hydrogen bonds into a three-dimensional network (Fig. 3).

### Experimental

A mixture of 5,6-dimethoxy(*E*)-2-(4-fluorobenzylidene)-2,3-dihydro-1*H*-indene-1-one (0.001 mol), ninhydrin (0.001 mol) and sarcosine (0.002 mol) (1:1:2) were dissolved in methanol (10 ml) and refluxed for 4 h. After completion of the reaction as evident from TLC, the excess solvent was evaporated slowly and the product was separated and recrystallized from methanol to reveal the title compound as yellow crystals.

### Refinement

O bound H atoms were located from a difference Fourier maps and freely refined. The remaining H atoms were positioned geometrically and refined using a riding model with C—H = 0.95–1.00 Å and *U*_iso_(H) = 1.2*U*_eq_(C) and 1.5*U*_eq_(*C*-methyl).

### Crystal data

**Table:**

C_29_H_24_FNO_5_·0.5CH_4_O	*F*(000) = 2104
*M**_r_* = 501.52	*D*_x_ = 1.390 Mg m^−^^3^
Monoclinic, *P*2_1_/*c*	Mo *K*α radiation, λ = 0.71073 Å
Hall symbol: -P 2ybc	Cell parameters from 9905 reflections
*a* = 14.6385 (6) Å	θ = 2.3–30.1°
*b* = 12.5099 (6) Å	µ = 0.10 mm^−^^1^
*c* = 26.2017 (10) Å	*T* = 100 K
β = 92.645 (1)°	Block, yellow
*V* = 4793.1 (4) Å^3^	0.44 × 0.21 × 0.15 mm
*Z* = 8	

### Data collection

**Table:**

Bruker APEX DUO CCD area-detector diffractometer	14094 independent reflections
Radiation source: fine-focus sealed tube	10283 reflections with *I* > 2σ(*I*)
Graphite monochromator	*R*_int_ = 0.046
φ and ω scans	θ_max_ = 30.2°, θ_min_ = 1.4°
Absorption correction: multi-scan (*SADABS*; Bruker, 2009)	*h* = −20→19
*T*_min_ = 0.957, *T*_max_ = 0.985	*k* = −17→17
53390 measured reflections	*l* = −37→37

### Refinement

**Table:**

Refinement on *F*^2^	Primary atom site location: structure-invariant direct methods
Least-squares matrix: full	Secondary atom site location: difference Fourier map
*R*[*F*^2^ > 2σ(*F*^2^)] = 0.049	Hydrogen site location: inferred from neighbouring sites
*wR*(*F*^2^) = 0.147	H atoms treated by a mixture of independent and constrained refinement
*S* = 1.04	*w* = 1/[σ^2^(*F*_o_^2^) + (0.0784*P*)^2^ + 0.9156*P*] where *P* = (*F*_o_^2^ + 2*F*_c_^2^)/3
14094 reflections	(Δ/σ)_max_ = 0.001
678 parameters	Δρ_max_ = 0.40 e Å^−^^3^
0 restraints	Δρ_min_ = −0.27 e Å^−^^3^

### Special details

**Table:**

Experimental. The crystal was placed in the cold stream of an Oxford Cryosystems Cobra open-flow nitrogen cryostat (Cosier & Glazer, 1986) operating at 100.0 (1) K.
Geometry. All e.s.d.'s (except the e.s.d. in the dihedral angle between two l.s. planes) are estimated using the full covariance matrix. The cell e.s.d.'s are taken into account individually in the estimation of e.s.d.'s in distances, angles and torsion angles; correlations between e.s.d.'s in cell parameters are only used when they are defined by crystal symmetry. An approximate (isotropic) treatment of cell e.s.d.'s is used for estimating e.s.d.'s involving l.s. planes.
Refinement. Refinement of *F*^2^ against ALL reflections. The weighted *R*-factor *wR* and goodness of fit *S* are based on *F*^2^, conventional *R*-factors *R* are based on *F*, with *F* set to zero for negative *F*^2^. The threshold expression of *F*^2^ > σ(*F*^2^) is used only for calculating *R*-factors(gt) *etc*. and is not relevant to the choice of reflections for refinement. *R*-factors based on *F*^2^ are statistically about twice as large as those based on *F*, and *R*- factors based on ALL data will be even larger.

### Fractional atomic coordinates and isotropic or equivalent isotropic displacement parameters (Å^2^)

**Table:**

	*x*	*y*	*z*	*U*_iso_*/*U*_eq_	
F1A	0.64124 (8)	0.50699 (10)	1.07056 (4)	0.0408 (3)	
O1A	0.57712 (7)	0.75263 (10)	0.83001 (4)	0.0224 (2)	
O2A	0.90976 (7)	0.94421 (10)	0.81133 (4)	0.0217 (2)	
O3A	0.92179 (7)	1.05466 (10)	0.89424 (4)	0.0230 (2)	
O4A	0.36455 (7)	0.82926 (10)	0.82207 (4)	0.0235 (2)	
O5A	0.52744 (8)	1.12958 (10)	0.87961 (4)	0.0297 (3)	
N1A	0.40617 (8)	0.96181 (12)	0.91618 (4)	0.0213 (3)	
C1A	0.60138 (9)	0.82167 (13)	0.86036 (5)	0.0169 (3)	
C2A	0.68848 (9)	0.87866 (12)	0.86443 (5)	0.0159 (3)	
C3A	0.75949 (9)	0.87575 (13)	0.83026 (5)	0.0166 (3)	
H3AA	0.7549	0.8325	0.8005	0.020*	
C4A	0.83602 (9)	0.93716 (13)	0.84103 (5)	0.0170 (3)	
C5A	0.84241 (9)	0.99947 (13)	0.88693 (5)	0.0179 (3)	
C6A	0.77115 (9)	1.00136 (13)	0.92012 (5)	0.0190 (3)	
H6AA	0.7756	1.0429	0.9505	0.023*	
C7A	0.69312 (9)	0.94131 (13)	0.90805 (5)	0.0168 (3)	
C8A	0.60674 (9)	0.93285 (14)	0.93741 (5)	0.0199 (3)	
H8AA	0.5820	1.0046	0.9449	0.024*	
H8AB	0.6183	0.8939	0.9699	0.024*	
C9A	0.54029 (9)	0.86988 (13)	0.90093 (5)	0.0177 (3)	
C10A	0.47557 (9)	0.78925 (13)	0.92612 (5)	0.0186 (3)	
H10A	0.4509	0.7414	0.8982	0.022*	
C11A	0.39533 (10)	0.85875 (14)	0.94223 (5)	0.0230 (3)	
H11A	0.3362	0.8252	0.9316	0.028*	
H11B	0.3976	0.8686	0.9798	0.028*	
C12A	0.46681 (9)	0.94658 (13)	0.87478 (5)	0.0188 (3)	
C13A	0.42549 (9)	0.89548 (13)	0.82479 (5)	0.0187 (3)	
C14A	0.46926 (9)	0.94764 (13)	0.78149 (5)	0.0192 (3)	
C15A	0.46305 (10)	0.92065 (14)	0.72959 (5)	0.0225 (3)	
H15A	0.4294	0.8600	0.7177	0.027*	
C16A	0.50824 (11)	0.98630 (15)	0.69640 (6)	0.0265 (3)	
H16A	0.5048	0.9707	0.6609	0.032*	
C17A	0.55860 (11)	1.07468 (15)	0.71386 (6)	0.0283 (4)	
H17A	0.5888	1.1177	0.6900	0.034*	
C18A	0.56576 (11)	1.10154 (14)	0.76558 (6)	0.0251 (3)	
H18A	0.6005	1.1614	0.7775	0.030*	
C19A	0.51950 (10)	1.03640 (13)	0.79895 (5)	0.0203 (3)	
C20A	0.50932 (10)	1.05031 (14)	0.85470 (5)	0.0213 (3)	
C21A	0.52098 (9)	0.71733 (13)	0.96626 (5)	0.0194 (3)	
C22A	0.56305 (10)	0.62396 (14)	0.95030 (5)	0.0228 (3)	
H22A	0.5630	0.6082	0.9148	0.027*	
C23A	0.60491 (11)	0.55361 (15)	0.98488 (6)	0.0280 (3)	
H23A	0.6338	0.4905	0.9735	0.034*	
C24A	0.60348 (11)	0.57771 (15)	1.03629 (6)	0.0264 (3)	
C25A	0.56456 (11)	0.66976 (15)	1.05385 (6)	0.0266 (3)	
H25A	0.5658	0.6853	1.0894	0.032*	
C26A	0.52338 (10)	0.73952 (14)	1.01870 (5)	0.0232 (3)	
H26A	0.4963	0.8035	1.0304	0.028*	
C27A	0.90161 (11)	0.88598 (15)	0.76404 (5)	0.0241 (3)	
H27A	0.9585	0.8927	0.7460	0.036*	
H27B	0.8508	0.9151	0.7427	0.036*	
H27C	0.8900	0.8104	0.7711	0.036*	
C28A	0.94184 (11)	1.09428 (14)	0.94499 (5)	0.0241 (3)	
H28A	1.0031	1.1262	0.9468	0.036*	
H28B	0.9396	1.0352	0.9694	0.036*	
H28C	0.8966	1.1485	0.9533	0.036*	
C29A	0.32106 (11)	1.01814 (16)	0.90408 (6)	0.0301 (4)	
H29A	0.3344	1.0873	0.8885	0.045*	
H29B	0.2885	1.0296	0.9355	0.045*	
H29C	0.2829	0.9754	0.8801	0.045*	
F1B	0.73958 (9)	0.84660 (11)	0.64602 (4)	0.0508 (3)	
O1B	0.70176 (7)	0.52113 (10)	0.86215 (4)	0.0209 (2)	
O2B	0.87193 (7)	0.80278 (10)	0.99446 (3)	0.0214 (2)	
O3B	1.01505 (7)	0.84762 (9)	0.94574 (4)	0.0201 (2)	
O4B	0.72154 (7)	0.27525 (10)	0.83132 (4)	0.0265 (2)	
O5B	1.01994 (7)	0.37946 (11)	0.87438 (4)	0.0271 (3)	
N1B	0.89700 (8)	0.33138 (11)	0.78465 (4)	0.0196 (3)	
C1B	0.78116 (9)	0.54550 (12)	0.85591 (4)	0.0152 (3)	
C2B	0.83736 (9)	0.62300 (12)	0.88429 (5)	0.0147 (3)	
C3B	0.81686 (9)	0.67684 (13)	0.92947 (5)	0.0163 (3)	
H3BA	0.7610	0.6642	0.9455	0.020*	
C4B	0.88014 (9)	0.74835 (12)	0.94969 (4)	0.0162 (3)	
C5B	0.96145 (9)	0.77097 (12)	0.92353 (5)	0.0163 (3)	
C6B	0.98171 (9)	0.71501 (12)	0.87953 (5)	0.0161 (3)	
H6BA	1.0368	0.7283	0.8628	0.019*	
C7B	0.91913 (9)	0.63893 (12)	0.86061 (5)	0.0147 (3)	
C8B	0.92686 (9)	0.56662 (12)	0.81472 (5)	0.0162 (3)	
H8BA	0.9838	0.5239	0.8176	0.019*	
H8BB	0.9264	0.6087	0.7827	0.019*	
C9B	0.84145 (9)	0.49360 (12)	0.81595 (5)	0.0152 (3)	
C10B	0.79019 (9)	0.47193 (13)	0.76358 (5)	0.0171 (3)	
H10B	0.7275	0.4467	0.7713	0.021*	
C11B	0.84021 (11)	0.37537 (15)	0.74183 (5)	0.0246 (3)	
H11C	0.7958	0.3213	0.7285	0.030*	
H11D	0.8789	0.3978	0.7137	0.030*	
C12B	0.86621 (9)	0.37560 (13)	0.83180 (5)	0.0168 (3)	
C13B	0.78331 (10)	0.31936 (13)	0.85524 (5)	0.0195 (3)	
C14B	0.79773 (10)	0.32678 (13)	0.91197 (5)	0.0217 (3)	
C15B	0.73581 (12)	0.30775 (15)	0.94960 (6)	0.0293 (4)	
H15B	0.6758	0.2824	0.9413	0.035*	
C16B	0.76572 (14)	0.32748 (16)	1.00007 (6)	0.0356 (4)	
H16B	0.7248	0.3165	1.0267	0.043*	
C17B	0.85401 (14)	0.36275 (16)	1.01225 (6)	0.0349 (4)	
H17B	0.8720	0.3763	1.0470	0.042*	
C18B	0.91653 (12)	0.37851 (15)	0.97473 (5)	0.0285 (4)	
H18B	0.9776	0.4002	0.9831	0.034*	
C19B	0.88584 (10)	0.36111 (13)	0.92418 (5)	0.0211 (3)	
C20B	0.93768 (10)	0.37345 (13)	0.87703 (5)	0.0190 (3)	
C21B	0.77768 (10)	0.57045 (13)	0.73034 (5)	0.0180 (3)	
C22B	0.70115 (10)	0.63458 (14)	0.73650 (5)	0.0215 (3)	
H22B	0.6576	0.6144	0.7605	0.026*	
C23B	0.68725 (12)	0.72781 (15)	0.70811 (6)	0.0289 (4)	
H23B	0.6345	0.7708	0.7121	0.035*	
C24B	0.75230 (13)	0.75584 (15)	0.67412 (6)	0.0332 (4)	
C25B	0.82992 (12)	0.69646 (16)	0.66743 (5)	0.0303 (4)	
H25B	0.8739	0.7187	0.6441	0.036*	
C26B	0.84227 (10)	0.60341 (15)	0.69561 (5)	0.0236 (3)	
H26B	0.8953	0.5612	0.6914	0.028*	
C27B	0.80001 (11)	0.76736 (16)	1.02551 (5)	0.0270 (4)	
H27D	0.8031	0.8065	1.0579	0.041*	
H27E	0.8069	0.6906	1.0322	0.041*	
H27F	0.7408	0.7807	1.0076	0.041*	
C28B	1.09083 (10)	0.88541 (14)	0.91793 (6)	0.0246 (3)	
H28D	1.1196	0.9460	0.9361	0.037*	
H28E	1.0690	0.9082	0.8837	0.037*	
H28F	1.1356	0.8278	0.9150	0.037*	
C29B	0.91046 (12)	0.21643 (14)	0.78356 (6)	0.0261 (3)	
H29D	0.9498	0.1948	0.8130	0.039*	
H29E	0.9395	0.1965	0.7520	0.039*	
H29F	0.8512	0.1804	0.7849	0.039*	
O6	0.22217 (10)	0.69772 (12)	0.86621 (5)	0.0390 (3)	
C30	0.21176 (13)	0.59596 (17)	0.84332 (7)	0.0352 (4)	
H30A	0.1591	0.5595	0.8572	0.053*	
H30B	0.2671	0.5535	0.8506	0.053*	
H30C	0.2019	0.6042	0.8063	0.053*	
H1O6	0.277 (2)	0.733 (3)	0.8553 (10)	0.072 (9)*	

### Atomic displacement parameters (Å^2^)

**Table:**

	*U*^11^	*U*^22^	*U*^33^	*U*^12^	*U*^13^	*U*^23^
F1A	0.0473 (6)	0.0336 (7)	0.0402 (5)	0.0073 (5)	−0.0137 (5)	0.0095 (5)
O1A	0.0207 (5)	0.0232 (6)	0.0233 (5)	−0.0028 (4)	0.0017 (4)	−0.0061 (4)
O2A	0.0180 (5)	0.0276 (7)	0.0198 (4)	−0.0052 (4)	0.0042 (3)	−0.0008 (4)
O3A	0.0200 (5)	0.0278 (7)	0.0209 (4)	−0.0085 (5)	−0.0032 (4)	−0.0018 (4)
O4A	0.0189 (5)	0.0248 (6)	0.0268 (5)	−0.0033 (5)	0.0000 (4)	0.0022 (4)
O5A	0.0339 (6)	0.0223 (7)	0.0331 (5)	−0.0023 (5)	0.0060 (5)	−0.0066 (5)
N1A	0.0189 (6)	0.0227 (7)	0.0229 (5)	0.0055 (5)	0.0067 (4)	0.0016 (5)
C1A	0.0153 (6)	0.0192 (8)	0.0163 (5)	0.0008 (6)	0.0012 (4)	−0.0002 (5)
C2A	0.0143 (6)	0.0167 (7)	0.0168 (5)	0.0009 (5)	0.0003 (4)	−0.0005 (5)
C3A	0.0166 (6)	0.0176 (7)	0.0157 (5)	0.0013 (5)	0.0007 (4)	−0.0012 (5)
C4A	0.0149 (6)	0.0189 (8)	0.0171 (5)	0.0003 (5)	0.0009 (4)	0.0017 (5)
C5A	0.0169 (6)	0.0175 (8)	0.0189 (5)	−0.0025 (6)	−0.0030 (5)	0.0011 (5)
C6A	0.0199 (6)	0.0189 (8)	0.0179 (5)	−0.0004 (6)	−0.0016 (5)	−0.0029 (5)
C7A	0.0154 (6)	0.0187 (8)	0.0162 (5)	0.0020 (6)	0.0005 (4)	−0.0005 (5)
C8A	0.0179 (6)	0.0231 (8)	0.0187 (5)	0.0001 (6)	0.0021 (5)	−0.0046 (5)
C9A	0.0147 (6)	0.0209 (8)	0.0178 (5)	0.0014 (6)	0.0031 (4)	−0.0014 (5)
C10A	0.0164 (6)	0.0212 (8)	0.0184 (5)	0.0000 (6)	0.0024 (4)	−0.0002 (5)
C11A	0.0195 (6)	0.0270 (9)	0.0230 (6)	0.0040 (6)	0.0063 (5)	0.0044 (6)
C12A	0.0165 (6)	0.0203 (8)	0.0199 (6)	0.0020 (6)	0.0041 (5)	0.0004 (5)
C13A	0.0156 (6)	0.0188 (8)	0.0218 (6)	0.0020 (6)	0.0022 (5)	0.0028 (5)
C14A	0.0166 (6)	0.0197 (8)	0.0215 (6)	0.0026 (6)	0.0023 (5)	0.0030 (5)
C15A	0.0214 (7)	0.0236 (9)	0.0226 (6)	0.0046 (6)	0.0006 (5)	0.0017 (6)
C16A	0.0268 (7)	0.0305 (10)	0.0226 (6)	0.0064 (7)	0.0040 (5)	0.0048 (6)
C17A	0.0262 (7)	0.0288 (10)	0.0305 (7)	0.0042 (7)	0.0089 (6)	0.0118 (7)
C18A	0.0228 (7)	0.0200 (9)	0.0328 (7)	0.0009 (6)	0.0041 (6)	0.0032 (6)
C19A	0.0179 (6)	0.0187 (8)	0.0245 (6)	0.0037 (6)	0.0030 (5)	0.0013 (6)
C20A	0.0182 (6)	0.0191 (8)	0.0266 (6)	0.0027 (6)	0.0036 (5)	−0.0004 (6)
C21A	0.0168 (6)	0.0210 (8)	0.0204 (6)	−0.0007 (6)	0.0021 (5)	0.0011 (5)
C22A	0.0210 (7)	0.0243 (9)	0.0232 (6)	−0.0001 (6)	0.0026 (5)	−0.0029 (6)
C23A	0.0248 (7)	0.0234 (9)	0.0356 (8)	0.0026 (7)	0.0002 (6)	−0.0019 (7)
C24A	0.0236 (7)	0.0252 (9)	0.0298 (7)	−0.0006 (7)	−0.0040 (6)	0.0069 (6)
C25A	0.0272 (7)	0.0306 (10)	0.0220 (6)	0.0000 (7)	0.0012 (5)	0.0016 (6)
C26A	0.0253 (7)	0.0244 (9)	0.0201 (6)	0.0029 (7)	0.0034 (5)	−0.0013 (6)
C27A	0.0239 (7)	0.0297 (9)	0.0193 (6)	−0.0036 (7)	0.0068 (5)	−0.0014 (6)
C28A	0.0241 (7)	0.0224 (9)	0.0249 (6)	−0.0032 (6)	−0.0077 (5)	−0.0046 (6)
C29A	0.0239 (7)	0.0332 (11)	0.0338 (8)	0.0120 (7)	0.0092 (6)	0.0071 (7)
F1B	0.0692 (8)	0.0313 (7)	0.0496 (6)	−0.0141 (6)	−0.0230 (6)	0.0227 (5)
O1B	0.0152 (4)	0.0251 (6)	0.0226 (4)	−0.0025 (4)	0.0032 (3)	−0.0027 (4)
O2B	0.0222 (5)	0.0258 (6)	0.0163 (4)	−0.0008 (5)	0.0024 (3)	−0.0082 (4)
O3B	0.0190 (5)	0.0203 (6)	0.0212 (4)	−0.0058 (4)	0.0014 (3)	−0.0047 (4)
O4B	0.0228 (5)	0.0242 (7)	0.0325 (5)	−0.0055 (5)	0.0006 (4)	−0.0008 (5)
O5B	0.0192 (5)	0.0313 (7)	0.0305 (5)	0.0025 (5)	−0.0009 (4)	−0.0025 (5)
N1B	0.0228 (6)	0.0196 (7)	0.0167 (5)	0.0028 (5)	0.0027 (4)	−0.0037 (5)
C1B	0.0147 (6)	0.0165 (7)	0.0146 (5)	0.0004 (5)	0.0011 (4)	0.0012 (5)
C2B	0.0150 (6)	0.0147 (7)	0.0144 (5)	−0.0001 (5)	0.0013 (4)	−0.0006 (5)
C3B	0.0147 (6)	0.0184 (8)	0.0159 (5)	0.0015 (5)	0.0025 (4)	−0.0008 (5)
C4B	0.0181 (6)	0.0165 (7)	0.0138 (5)	0.0024 (5)	0.0008 (4)	−0.0020 (5)
C5B	0.0167 (6)	0.0156 (7)	0.0164 (5)	−0.0018 (5)	−0.0015 (4)	−0.0001 (5)
C6B	0.0159 (6)	0.0167 (7)	0.0159 (5)	−0.0010 (5)	0.0026 (4)	0.0000 (5)
C7B	0.0149 (6)	0.0149 (7)	0.0142 (5)	0.0001 (5)	0.0014 (4)	0.0004 (5)
C8B	0.0156 (6)	0.0172 (7)	0.0159 (5)	−0.0025 (5)	0.0032 (4)	−0.0024 (5)
C9B	0.0150 (6)	0.0153 (7)	0.0155 (5)	−0.0011 (5)	0.0019 (4)	−0.0022 (5)
C10B	0.0184 (6)	0.0170 (8)	0.0160 (5)	−0.0020 (6)	0.0004 (4)	−0.0026 (5)
C11B	0.0308 (8)	0.0253 (9)	0.0175 (6)	0.0058 (7)	−0.0025 (5)	−0.0066 (6)
C12B	0.0165 (6)	0.0176 (8)	0.0165 (5)	−0.0003 (6)	0.0018 (4)	−0.0018 (5)
C13B	0.0188 (6)	0.0177 (8)	0.0221 (6)	0.0013 (6)	0.0033 (5)	0.0010 (5)
C14B	0.0262 (7)	0.0172 (8)	0.0223 (6)	0.0033 (6)	0.0057 (5)	0.0027 (6)
C15B	0.0337 (8)	0.0252 (9)	0.0298 (7)	0.0049 (7)	0.0115 (6)	0.0092 (7)
C16B	0.0518 (11)	0.0295 (10)	0.0269 (7)	0.0137 (9)	0.0181 (7)	0.0110 (7)
C17B	0.0586 (12)	0.0276 (10)	0.0184 (6)	0.0126 (9)	0.0022 (7)	0.0034 (6)
C18B	0.0405 (9)	0.0243 (9)	0.0200 (6)	0.0078 (7)	−0.0044 (6)	0.0006 (6)
C19B	0.0289 (7)	0.0162 (8)	0.0183 (6)	0.0059 (6)	0.0014 (5)	0.0019 (5)
C20B	0.0189 (6)	0.0180 (8)	0.0200 (6)	0.0019 (6)	0.0002 (5)	−0.0027 (5)
C21B	0.0209 (6)	0.0181 (8)	0.0148 (5)	−0.0034 (6)	−0.0017 (4)	−0.0022 (5)
C22B	0.0224 (7)	0.0202 (8)	0.0215 (6)	−0.0024 (6)	−0.0019 (5)	−0.0016 (6)
C23B	0.0337 (8)	0.0184 (9)	0.0334 (7)	0.0004 (7)	−0.0112 (6)	−0.0002 (6)
C24B	0.0471 (10)	0.0220 (9)	0.0288 (7)	−0.0124 (8)	−0.0166 (7)	0.0098 (7)
C25B	0.0365 (9)	0.0349 (11)	0.0191 (6)	−0.0173 (8)	−0.0046 (6)	0.0044 (6)
C26B	0.0242 (7)	0.0312 (10)	0.0155 (5)	−0.0076 (7)	−0.0002 (5)	−0.0017 (6)
C27B	0.0257 (7)	0.0367 (11)	0.0193 (6)	−0.0002 (7)	0.0067 (5)	−0.0064 (6)
C28B	0.0212 (7)	0.0237 (9)	0.0291 (7)	−0.0072 (6)	0.0040 (5)	−0.0031 (6)
C29B	0.0307 (8)	0.0212 (9)	0.0267 (7)	0.0043 (7)	0.0023 (6)	−0.0046 (6)
O6	0.0455 (8)	0.0322 (8)	0.0409 (7)	−0.0065 (6)	0.0185 (6)	−0.0034 (6)
C30	0.0367 (9)	0.0344 (11)	0.0346 (8)	0.0034 (8)	0.0009 (7)	−0.0008 (8)

### Geometric parameters (Å, º)

**Table:**

F1A—C24A	1.3597 (19)	O2B—C27B	1.4302 (18)
O1A—C1A	1.2158 (18)	O3B—C5B	1.3534 (17)
O2A—C4A	1.3624 (16)	O3B—C28B	1.4351 (18)
O2A—C27A	1.4376 (18)	O4B—C13B	1.2093 (18)
O3A—C5A	1.3575 (17)	O5B—C20B	1.2116 (18)
O3A—C28A	1.4366 (17)	N1B—C12B	1.4446 (17)
O4A—C13A	1.2170 (19)	N1B—C29B	1.452 (2)
O5A—C20A	1.210 (2)	N1B—C11B	1.4719 (18)
N1A—C12A	1.4455 (17)	C1B—C2B	1.4538 (19)
N1A—C29A	1.453 (2)	C1B—C9B	1.5433 (18)
N1A—C11A	1.471 (2)	C2B—C7B	1.3877 (18)
C1A—C2A	1.4604 (19)	C2B—C3B	1.4064 (18)
C1A—C9A	1.5432 (18)	C3B—C4B	1.376 (2)
C2A—C7A	1.3848 (19)	C3B—H3BA	0.9500
C2A—C3A	1.4034 (18)	C4B—C5B	1.4290 (19)
C3A—C4A	1.377 (2)	C5B—C6B	1.3924 (18)
C3A—H3AA	0.9500	C6B—C7B	1.396 (2)
C4A—C5A	1.4326 (19)	C6B—H6BA	0.9500
C5A—C6A	1.3888 (19)	C7B—C8B	1.5132 (18)
C6A—C7A	1.391 (2)	C8B—C9B	1.5499 (19)
C6A—H6AA	0.9500	C8B—H8BA	0.9900
C7A—C8A	1.5138 (19)	C8B—H8BB	0.9900
C8A—C9A	1.547 (2)	C9B—C10B	1.5570 (17)
C8A—H8AA	0.9900	C9B—C12B	1.571 (2)
C8A—H8AB	0.9900	C10B—C21B	1.515 (2)
C9A—C10A	1.552 (2)	C10B—C11B	1.536 (2)
C9A—C12A	1.575 (2)	C10B—H10B	1.0000
C10A—C21A	1.514 (2)	C11B—H11C	0.9900
C10A—C11A	1.536 (2)	C11B—H11D	0.9900
C10A—H10A	1.0000	C12B—C20B	1.5446 (18)
C11A—H11A	0.9900	C12B—C13B	1.554 (2)
C11A—H11B	0.9900	C13B—C14B	1.4944 (19)
C12A—C20A	1.542 (2)	C14B—C19B	1.383 (2)
C12A—C13A	1.555 (2)	C14B—C15B	1.390 (2)
C13A—C14A	1.4801 (19)	C15B—C16B	1.396 (2)
C14A—C19A	1.397 (2)	C15B—H15B	0.9500
C14A—C15A	1.4000 (19)	C16B—C17B	1.389 (3)
C15A—C16A	1.386 (2)	C16B—H16B	0.9500
C15A—H15A	0.9500	C17B—C18B	1.388 (2)
C16A—C17A	1.394 (3)	C17B—H17B	0.9500
C16A—H16A	0.9500	C18B—C19B	1.3959 (19)
C17A—C18A	1.395 (2)	C18B—H18B	0.9500
C17A—H17A	0.9500	C19B—C20B	1.4874 (19)
C18A—C19A	1.393 (2)	C21B—C22B	1.393 (2)
C18A—H18A	0.9500	C21B—C26B	1.404 (2)
C19A—C20A	1.4853 (19)	C22B—C23B	1.393 (2)
C21A—C22A	1.394 (2)	C22B—H22B	0.9500
C21A—C26A	1.4004 (19)	C23B—C24B	1.379 (3)
C22A—C23A	1.385 (2)	C23B—H23B	0.9500
C22A—H22A	0.9500	C24B—C25B	1.375 (3)
C23A—C24A	1.382 (2)	C25B—C26B	1.386 (2)
C23A—H23A	0.9500	C25B—H25B	0.9500
C24A—C25A	1.373 (3)	C26B—H26B	0.9500
C25A—C26A	1.386 (2)	C27B—H27D	0.9800
C25A—H25A	0.9500	C27B—H27E	0.9800
C26A—H26A	0.9500	C27B—H27F	0.9800
C27A—H27A	0.9800	C28B—H28D	0.9800
C27A—H27B	0.9800	C28B—H28E	0.9800
C27A—H27C	0.9800	C28B—H28F	0.9800
C28A—H28A	0.9800	C29B—H29D	0.9800
C28A—H28B	0.9800	C29B—H29E	0.9800
C28A—H28C	0.9800	C29B—H29F	0.9800
C29A—H29A	0.9800	O6—C30	1.412 (2)
C29A—H29B	0.9800	O6—H1O6	0.97 (3)
C29A—H29C	0.9800	C30—H30A	0.9800
F1B—C24B	1.361 (2)	C30—H30B	0.9800
O1B—C1B	1.2199 (16)	C30—H30C	0.9800
O2B—C4B	1.3666 (15)		
			
C4A—O2A—C27A	115.02 (11)	C29B—N1B—C11B	115.32 (12)
C5A—O3A—C28A	116.30 (11)	O1B—C1B—C2B	128.19 (12)
C12A—N1A—C29A	116.75 (12)	O1B—C1B—C9B	124.33 (13)
C12A—N1A—C11A	108.41 (12)	C2B—C1B—C9B	107.44 (11)
C29A—N1A—C11A	114.61 (13)	C7B—C2B—C3B	121.97 (12)
O1A—C1A—C2A	128.48 (12)	C7B—C2B—C1B	110.27 (11)
O1A—C1A—C9A	124.55 (13)	C3B—C2B—C1B	127.75 (12)
C2A—C1A—C9A	106.89 (12)	C4B—C3B—C2B	117.99 (12)
C7A—C2A—C3A	122.12 (13)	C4B—C3B—H3BA	121.0
C7A—C2A—C1A	110.24 (12)	C2B—C3B—H3BA	121.0
C3A—C2A—C1A	127.63 (12)	O2B—C4B—C3B	124.76 (12)
C4A—C3A—C2A	118.32 (12)	O2B—C4B—C5B	114.92 (12)
C4A—C3A—H3AA	120.8	C3B—C4B—C5B	120.32 (12)
C2A—C3A—H3AA	120.8	O3B—C5B—C6B	124.81 (12)
O2A—C4A—C3A	125.29 (12)	O3B—C5B—C4B	114.41 (11)
O2A—C4A—C5A	114.93 (12)	C6B—C5B—C4B	120.77 (13)
C3A—C4A—C5A	119.78 (12)	C5B—C6B—C7B	118.38 (12)
O3A—C5A—C6A	124.46 (13)	C5B—C6B—H6BA	120.8
O3A—C5A—C4A	114.67 (12)	C7B—C6B—H6BA	120.8
C6A—C5A—C4A	120.87 (13)	C2B—C7B—C6B	120.30 (12)
C5A—C6A—C7A	118.75 (13)	C2B—C7B—C8B	111.54 (12)
C5A—C6A—H6AA	120.6	C6B—C7B—C8B	128.16 (12)
C7A—C6A—H6AA	120.6	C7B—C8B—C9B	104.23 (10)
C2A—C7A—C6A	120.11 (12)	C7B—C8B—H8BA	110.9
C2A—C7A—C8A	111.69 (12)	C9B—C8B—H8BA	110.9
C6A—C7A—C8A	128.20 (12)	C7B—C8B—H8BB	110.9
C7A—C8A—C9A	103.75 (10)	C9B—C8B—H8BB	110.9
C7A—C8A—H8AA	111.0	H8BA—C8B—H8BB	108.9
C9A—C8A—H8AA	111.0	C1B—C9B—C8B	104.67 (11)
C7A—C8A—H8AB	111.0	C1B—C9B—C10B	113.81 (11)
C9A—C8A—H8AB	111.0	C8B—C9B—C10B	116.14 (11)
H8AA—C8A—H8AB	109.0	C1B—C9B—C12B	110.37 (11)
C1A—C9A—C8A	104.88 (11)	C8B—C9B—C12B	112.50 (11)
C1A—C9A—C10A	115.06 (13)	C10B—C9B—C12B	99.51 (11)
C8A—C9A—C10A	116.55 (11)	C21B—C10B—C11B	118.27 (11)
C1A—C9A—C12A	110.14 (10)	C21B—C10B—C9B	113.91 (12)
C8A—C9A—C12A	110.85 (13)	C11B—C10B—C9B	104.20 (11)
C10A—C9A—C12A	99.37 (11)	C21B—C10B—H10B	106.6
C21A—C10A—C11A	117.53 (11)	C11B—C10B—H10B	106.6
C21A—C10A—C9A	115.12 (11)	C9B—C10B—H10B	106.6
C11A—C10A—C9A	103.80 (13)	N1B—C11B—C10B	105.86 (11)
C21A—C10A—H10A	106.5	N1B—C11B—H11C	110.6
C11A—C10A—H10A	106.5	C10B—C11B—H11C	110.6
C9A—C10A—H10A	106.5	N1B—C11B—H11D	110.6
N1A—C11A—C10A	105.57 (11)	C10B—C11B—H11D	110.6
N1A—C11A—H11A	110.6	H11C—C11B—H11D	108.7
C10A—C11A—H11A	110.6	N1B—C12B—C20B	114.99 (11)
N1A—C11A—H11B	110.6	N1B—C12B—C13B	116.42 (12)
C10A—C11A—H11B	110.6	C20B—C12B—C13B	101.69 (11)
H11A—C11A—H11B	108.8	N1B—C12B—C9B	102.18 (10)
N1A—C12A—C20A	114.62 (13)	C20B—C12B—C9B	111.04 (12)
N1A—C12A—C13A	117.24 (12)	C13B—C12B—C9B	110.80 (11)
C20A—C12A—C13A	101.88 (11)	O4B—C13B—C14B	127.71 (14)
N1A—C12A—C9A	100.64 (10)	O4B—C13B—C12B	125.50 (12)
C20A—C12A—C9A	112.52 (11)	C14B—C13B—C12B	106.74 (12)
C13A—C12A—C9A	110.33 (12)	C19B—C14B—C15B	121.37 (14)
O4A—C13A—C14A	126.67 (13)	C19B—C14B—C13B	109.69 (12)
O4A—C13A—C12A	125.80 (12)	C15B—C14B—C13B	128.90 (15)
C14A—C13A—C12A	107.41 (12)	C14B—C15B—C16B	117.09 (17)
C19A—C14A—C15A	121.28 (14)	C14B—C15B—H15B	121.5
C19A—C14A—C13A	109.84 (12)	C16B—C15B—H15B	121.5
C15A—C14A—C13A	128.85 (15)	C17B—C16B—C15B	121.47 (15)
C16A—C15A—C14A	117.07 (15)	C17B—C16B—H16B	119.3
C16A—C15A—H15A	121.5	C15B—C16B—H16B	119.3
C14A—C15A—H15A	121.5	C18B—C17B—C16B	121.29 (15)
C15A—C16A—C17A	121.62 (14)	C18B—C17B—H17B	119.4
C15A—C16A—H16A	119.2	C16B—C17B—H17B	119.4
C17A—C16A—H16A	119.2	C17B—C18B—C19B	117.14 (16)
C16A—C17A—C18A	121.63 (15)	C17B—C18B—H18B	121.4
C16A—C17A—H17A	119.2	C19B—C18B—H18B	121.4
C18A—C17A—H17A	119.2	C14B—C19B—C18B	121.59 (14)
C19A—C18A—C17A	116.85 (16)	C14B—C19B—C20B	110.25 (12)
C19A—C18A—H18A	121.6	C18B—C19B—C20B	128.15 (15)
C17A—C18A—H18A	121.6	O5B—C20B—C19B	126.90 (13)
C18A—C19A—C14A	121.54 (13)	O5B—C20B—C12B	126.49 (12)
C18A—C19A—C20A	128.48 (15)	C19B—C20B—C12B	106.60 (11)
C14A—C19A—C20A	109.87 (13)	C22B—C21B—C26B	118.36 (15)
O5A—C20A—C19A	126.67 (15)	C22B—C21B—C10B	118.58 (12)
O5A—C20A—C12A	125.97 (13)	C26B—C21B—C10B	122.96 (14)
C19A—C20A—C12A	107.30 (13)	C23B—C22B—C21B	121.25 (15)
C22A—C21A—C26A	117.97 (14)	C23B—C22B—H22B	119.4
C22A—C21A—C10A	118.39 (12)	C21B—C22B—H22B	119.4
C26A—C21A—C10A	123.64 (14)	C24B—C23B—C22B	118.00 (17)
C23A—C22A—C21A	121.63 (14)	C24B—C23B—H23B	121.0
C23A—C22A—H22A	119.2	C22B—C23B—H23B	121.0
C21A—C22A—H22A	119.2	F1B—C24B—C25B	118.34 (17)
C24A—C23A—C22A	118.22 (16)	F1B—C24B—C23B	118.70 (18)
C24A—C23A—H23A	120.9	C25B—C24B—C23B	122.96 (16)
C22A—C23A—H23A	120.9	C24B—C25B—C26B	118.30 (15)
F1A—C24A—C25A	119.12 (14)	C24B—C25B—H25B	120.8
F1A—C24A—C23A	118.55 (16)	C26B—C25B—H25B	120.8
C25A—C24A—C23A	122.33 (15)	C25B—C26B—C21B	121.11 (16)
C24A—C25A—C26A	118.66 (14)	C25B—C26B—H26B	119.4
C24A—C25A—H25A	120.7	C21B—C26B—H26B	119.4
C26A—C25A—H25A	120.7	O2B—C27B—H27D	109.5
C25A—C26A—C21A	121.16 (15)	O2B—C27B—H27E	109.5
C25A—C26A—H26A	119.4	H27D—C27B—H27E	109.5
C21A—C26A—H26A	119.4	O2B—C27B—H27F	109.5
O2A—C27A—H27A	109.5	H27D—C27B—H27F	109.5
O2A—C27A—H27B	109.5	H27E—C27B—H27F	109.5
H27A—C27A—H27B	109.5	O3B—C28B—H28D	109.5
O2A—C27A—H27C	109.5	O3B—C28B—H28E	109.5
H27A—C27A—H27C	109.5	H28D—C28B—H28E	109.5
H27B—C27A—H27C	109.5	O3B—C28B—H28F	109.5
O3A—C28A—H28A	109.5	H28D—C28B—H28F	109.5
O3A—C28A—H28B	109.5	H28E—C28B—H28F	109.5
H28A—C28A—H28B	109.5	N1B—C29B—H29D	109.5
O3A—C28A—H28C	109.5	N1B—C29B—H29E	109.5
H28A—C28A—H28C	109.5	H29D—C29B—H29E	109.5
H28B—C28A—H28C	109.5	N1B—C29B—H29F	109.5
N1A—C29A—H29A	109.5	H29D—C29B—H29F	109.5
N1A—C29A—H29B	109.5	H29E—C29B—H29F	109.5
H29A—C29A—H29B	109.5	C30—O6—H1O6	111.3 (18)
N1A—C29A—H29C	109.5	O6—C30—H30A	109.5
H29A—C29A—H29C	109.5	O6—C30—H30B	109.5
H29B—C29A—H29C	109.5	H30A—C30—H30B	109.5
C4B—O2B—C27B	115.48 (12)	O6—C30—H30C	109.5
C5B—O3B—C28B	117.48 (11)	H30A—C30—H30C	109.5
C12B—N1B—C29B	116.36 (12)	H30B—C30—H30C	109.5
C12B—N1B—C11B	108.74 (11)		
			
O1A—C1A—C2A—C7A	−172.47 (15)	O1B—C1B—C2B—C7B	−171.09 (14)
C9A—C1A—C2A—C7A	10.63 (16)	C9B—C1B—C2B—C7B	10.90 (15)
O1A—C1A—C2A—C3A	8.6 (3)	O1B—C1B—C2B—C3B	9.2 (2)
C9A—C1A—C2A—C3A	−168.33 (14)	C9B—C1B—C2B—C3B	−168.79 (14)
C7A—C2A—C3A—C4A	0.3 (2)	C7B—C2B—C3B—C4B	1.0 (2)
C1A—C2A—C3A—C4A	179.15 (14)	C1B—C2B—C3B—C4B	−179.30 (14)
C27A—O2A—C4A—C3A	3.5 (2)	C27B—O2B—C4B—C3B	11.1 (2)
C27A—O2A—C4A—C5A	−177.05 (13)	C27B—O2B—C4B—C5B	−169.22 (13)
C2A—C3A—C4A—O2A	−178.90 (13)	C2B—C3B—C4B—O2B	−176.79 (13)
C2A—C3A—C4A—C5A	1.7 (2)	C2B—C3B—C4B—C5B	3.6 (2)
C28A—O3A—C5A—C6A	16.5 (2)	C28B—O3B—C5B—C6B	10.0 (2)
C28A—O3A—C5A—C4A	−163.83 (13)	C28B—O3B—C5B—C4B	−171.30 (13)
O2A—C4A—C5A—O3A	−1.07 (19)	O2B—C4B—C5B—O3B	−3.72 (18)
C3A—C4A—C5A—O3A	178.39 (13)	C3B—C4B—C5B—O3B	175.95 (13)
O2A—C4A—C5A—C6A	178.61 (13)	O2B—C4B—C5B—C6B	175.04 (13)
C3A—C4A—C5A—C6A	−1.9 (2)	C3B—C4B—C5B—C6B	−5.3 (2)
O3A—C5A—C6A—C7A	179.75 (14)	O3B—C5B—C6B—C7B	−179.17 (13)
C4A—C5A—C6A—C7A	0.1 (2)	C4B—C5B—C6B—C7B	2.2 (2)
C3A—C2A—C7A—C6A	−2.1 (2)	C3B—C2B—C7B—C6B	−4.1 (2)
C1A—C2A—C7A—C6A	178.83 (13)	C1B—C2B—C7B—C6B	176.18 (13)
C3A—C2A—C7A—C8A	178.17 (13)	C3B—C2B—C7B—C8B	176.41 (13)
C1A—C2A—C7A—C8A	−0.85 (17)	C1B—C2B—C7B—C8B	−3.30 (16)
C5A—C6A—C7A—C2A	1.9 (2)	C5B—C6B—C7B—C2B	2.4 (2)
C5A—C6A—C7A—C8A	−178.49 (14)	C5B—C6B—C7B—C8B	−178.24 (14)
C2A—C7A—C8A—C9A	−9.12 (17)	C2B—C7B—C8B—C9B	−5.56 (15)
C6A—C7A—C8A—C9A	171.23 (15)	C6B—C7B—C8B—C9B	175.01 (14)
O1A—C1A—C9A—C8A	167.21 (15)	O1B—C1B—C9B—C8B	168.11 (13)
C2A—C1A—C9A—C8A	−15.74 (15)	C2B—C1B—C9B—C8B	−13.78 (14)
O1A—C1A—C9A—C10A	37.81 (19)	O1B—C1B—C9B—C10B	40.27 (19)
C2A—C1A—C9A—C10A	−145.14 (12)	C2B—C1B—C9B—C10B	−141.62 (12)
O1A—C1A—C9A—C12A	−73.47 (19)	O1B—C1B—C9B—C12B	−70.62 (16)
C2A—C1A—C9A—C12A	103.58 (13)	C2B—C1B—C9B—C12B	107.49 (12)
C7A—C8A—C9A—C1A	14.71 (15)	C7B—C8B—C9B—C1B	11.48 (14)
C7A—C8A—C9A—C10A	143.22 (13)	C7B—C8B—C9B—C10B	137.88 (12)
C7A—C8A—C9A—C12A	−104.13 (13)	C7B—C8B—C9B—C12B	−108.38 (12)
C1A—C9A—C10A—C21A	77.09 (15)	C1B—C9B—C10B—C21B	78.01 (15)
C8A—C9A—C10A—C21A	−46.31 (18)	C8B—C9B—C10B—C21B	−43.66 (17)
C12A—C9A—C10A—C21A	−165.37 (12)	C12B—C9B—C10B—C21B	−164.63 (11)
C1A—C9A—C10A—C11A	−153.05 (11)	C1B—C9B—C10B—C11B	−151.70 (13)
C8A—C9A—C10A—C11A	83.55 (14)	C8B—C9B—C10B—C11B	86.63 (15)
C12A—C9A—C10A—C11A	−35.51 (13)	C12B—C9B—C10B—C11B	−34.33 (13)
C12A—N1A—C11A—C10A	17.98 (15)	C12B—N1B—C11B—C10B	15.16 (17)
C29A—N1A—C11A—C10A	150.38 (13)	C29B—N1B—C11B—C10B	147.94 (13)
C21A—C10A—C11A—N1A	141.21 (13)	C21B—C10B—C11B—N1B	141.35 (13)
C9A—C10A—C11A—N1A	12.82 (14)	C9B—C10B—C11B—N1B	13.70 (16)
C29A—N1A—C12A—C20A	67.19 (18)	C29B—N1B—C12B—C20B	70.02 (17)
C11A—N1A—C12A—C20A	−161.56 (12)	C11B—N1B—C12B—C20B	−157.75 (13)
C29A—N1A—C12A—C13A	−52.2 (2)	C29B—N1B—C12B—C13B	−48.74 (17)
C11A—N1A—C12A—C13A	79.06 (15)	C11B—N1B—C12B—C13B	83.49 (16)
C29A—N1A—C12A—C9A	−171.83 (14)	C29B—N1B—C12B—C9B	−169.60 (12)
C11A—N1A—C12A—C9A	−40.58 (14)	C11B—N1B—C12B—C9B	−37.37 (15)
C1A—C9A—C12A—N1A	167.34 (12)	C1B—C9B—C12B—N1B	163.49 (10)
C8A—C9A—C12A—N1A	−77.04 (14)	C8B—C9B—C12B—N1B	−80.01 (12)
C10A—C9A—C12A—N1A	46.16 (13)	C10B—C9B—C12B—N1B	43.56 (12)
C1A—C9A—C12A—C20A	−70.19 (15)	C1B—C9B—C12B—C20B	−73.41 (13)
C8A—C9A—C12A—C20A	45.42 (14)	C8B—C9B—C12B—C20B	43.09 (14)
C10A—C9A—C12A—C20A	168.62 (11)	C10B—C9B—C12B—C20B	166.66 (10)
C1A—C9A—C12A—C13A	42.84 (16)	C1B—C9B—C12B—C13B	38.82 (14)
C8A—C9A—C12A—C13A	158.46 (11)	C8B—C9B—C12B—C13B	155.32 (10)
C10A—C9A—C12A—C13A	−78.34 (13)	C10B—C9B—C12B—C13B	−81.11 (12)
N1A—C12A—C13A—O4A	−32.5 (2)	N1B—C12B—C13B—O4B	−31.6 (2)
C20A—C12A—C13A—O4A	−158.50 (15)	C20B—C12B—C13B—O4B	−157.37 (16)
C9A—C12A—C13A—O4A	81.81 (18)	C9B—C12B—C13B—O4B	84.55 (18)
N1A—C12A—C13A—C14A	143.58 (13)	N1B—C12B—C13B—C14B	145.98 (13)
C20A—C12A—C13A—C14A	17.63 (14)	C20B—C12B—C13B—C14B	20.21 (15)
C9A—C12A—C13A—C14A	−102.07 (14)	C9B—C12B—C13B—C14B	−97.87 (13)
O4A—C13A—C14A—C19A	165.60 (15)	O4B—C13B—C14B—C19B	165.96 (17)
C12A—C13A—C14A—C19A	−10.48 (16)	C12B—C13B—C14B—C19B	−11.55 (18)
O4A—C13A—C14A—C15A	−12.3 (3)	O4B—C13B—C14B—C15B	−16.3 (3)
C12A—C13A—C14A—C15A	171.57 (15)	C12B—C13B—C14B—C15B	166.19 (17)
C19A—C14A—C15A—C16A	−0.5 (2)	C19B—C14B—C15B—C16B	1.6 (3)
C13A—C14A—C15A—C16A	177.23 (15)	C13B—C14B—C15B—C16B	−175.89 (17)
C14A—C15A—C16A—C17A	0.7 (2)	C14B—C15B—C16B—C17B	−1.1 (3)
C15A—C16A—C17A—C18A	−0.2 (3)	C15B—C16B—C17B—C18B	−0.8 (3)
C16A—C17A—C18A—C19A	−0.6 (2)	C16B—C17B—C18B—C19B	2.2 (3)
C17A—C18A—C19A—C14A	0.8 (2)	C15B—C14B—C19B—C18B	−0.2 (3)
C17A—C18A—C19A—C20A	−174.99 (15)	C13B—C14B—C19B—C18B	177.76 (15)
C15A—C14A—C19A—C18A	−0.2 (2)	C15B—C14B—C19B—C20B	179.10 (15)
C13A—C14A—C19A—C18A	−178.36 (14)	C13B—C14B—C19B—C20B	−2.96 (19)
C15A—C14A—C19A—C20A	176.23 (13)	C17B—C18B—C19B—C14B	−1.8 (3)
C13A—C14A—C19A—C20A	−1.90 (17)	C17B—C18B—C19B—C20B	179.11 (17)
C18A—C19A—C20A—O5A	12.4 (3)	C14B—C19B—C20B—O5B	−162.86 (17)
C14A—C19A—C20A—O5A	−163.70 (15)	C18B—C19B—C20B—O5B	16.4 (3)
C18A—C19A—C20A—C12A	−170.24 (15)	C14B—C19B—C20B—C12B	16.38 (18)
C14A—C19A—C20A—C12A	13.61 (16)	C18B—C19B—C20B—C12B	−164.40 (16)
N1A—C12A—C20A—O5A	31.0 (2)	N1B—C12B—C20B—O5B	30.7 (2)
C13A—C12A—C20A—O5A	158.67 (15)	C13B—C12B—C20B—O5B	157.38 (17)
C9A—C12A—C20A—O5A	−83.19 (18)	C9B—C12B—C20B—O5B	−84.71 (19)
N1A—C12A—C20A—C19A	−146.33 (12)	N1B—C12B—C20B—C19B	−148.57 (13)
C13A—C12A—C20A—C19A	−18.67 (14)	C13B—C12B—C20B—C19B	−21.86 (16)
C9A—C12A—C20A—C19A	99.47 (13)	C9B—C12B—C20B—C19B	96.05 (14)
C11A—C10A—C21A—C22A	152.37 (15)	C11B—C10B—C21B—C22B	149.87 (13)
C9A—C10A—C21A—C22A	−84.84 (17)	C9B—C10B—C21B—C22B	−87.23 (15)
C11A—C10A—C21A—C26A	−27.8 (2)	C11B—C10B—C21B—C26B	−33.80 (19)
C9A—C10A—C21A—C26A	94.95 (17)	C9B—C10B—C21B—C26B	89.10 (16)
C26A—C21A—C22A—C23A	1.1 (2)	C26B—C21B—C22B—C23B	1.6 (2)
C10A—C21A—C22A—C23A	−179.15 (14)	C10B—C21B—C22B—C23B	178.14 (13)
C21A—C22A—C23A—C24A	0.5 (2)	C21B—C22B—C23B—C24B	−0.9 (2)
C22A—C23A—C24A—F1A	177.43 (15)	C22B—C23B—C24B—F1B	179.86 (14)
C22A—C23A—C24A—C25A	−1.9 (3)	C22B—C23B—C24B—C25B	−0.5 (2)
F1A—C24A—C25A—C26A	−177.74 (15)	F1B—C24B—C25B—C26B	−179.24 (14)
C23A—C24A—C25A—C26A	1.6 (3)	C23B—C24B—C25B—C26B	1.1 (2)
C24A—C25A—C26A—C21A	0.1 (2)	C24B—C25B—C26B—C21B	−0.3 (2)
C22A—C21A—C26A—C25A	−1.4 (2)	C22B—C21B—C26B—C25B	−1.0 (2)
C10A—C21A—C26A—C25A	178.84 (15)	C10B—C21B—C26B—C25B	−177.34 (13)

### Hydrogen-bond geometry (Å, º)

**Table:**

*D*—H···*A*	*D*—H	H···*A*	*D*···*A*	*D*—H···*A*
O6—H1*O*6···O4*A*	0.97 (3)	1.99 (3)	2.9346 (18)	164 (3)
C8*A*—H8*AA*···O5*A*	0.99	2.43	3.088 (2)	124
C8*B*—H8*BA*···O5*B*	0.99	2.38	3.0960 (19)	128
C26*A*—H26*A*···O5*A*^i^	0.95	2.54	3.2430 (18)	131
C27*A*—H27*A*···N1*B*^ii^	0.98	2.42	3.337 (2)	155
C28*A*—H28*A*···O2*B*^iii^	0.98	2.50	3.3478 (19)	145
C28*B*—H28*F*···O6^iv^	0.98	2.46	3.360 (2)	153
C30—H30*B*···F1*A*^v^	0.98	2.53	3.307 (2)	136

Symmetry codes: (i) −*x*+1, −*y*+2, −*z*+2; (ii) −*x*+2, *y*+1/2, −*z*+3/2; (iii) −*x*+2, −*y*+2, −*z*+2; (iv) *x*+1, *y*, *z*; (v) −*x*+1, −*y*+1, −*z*+2.
